# Vitamin-Mediated Regulation of Intestinal Immunity

**DOI:** 10.3389/fimmu.2013.00189

**Published:** 2013-07-11

**Authors:** Jun Kunisawa, Hiroshi Kiyono

**Affiliations:** ^1^Laboratory of Vaccine Materials, National Institute of Biomedical Innovation, Osaka, Japan; ^2^Division of Mucosal Immunology, Department of Microbiology and Immunology, The Institute of Medical Science, The University of Tokyo, Tokyo, Japan; ^3^International Research and Development Center for Mucosal Vaccines, The Institute of Medical Science, The University of Tokyo, Tokyo, Japan; ^4^Core Research for Evolutional Science and Technology (CREST), Japan Science and Technology Agency, Tokyo, Japan; ^5^Graduate School of Pharmaceutical Sciences, Osaka University, Osaka, Japan; ^6^Department of Medical Genome Science, Graduate School of Frontier Science, The University of Tokyo, Chiba, Japan; ^7^Graduate School of Medicine, The University of Tokyo, Tokyo, Japan

**Keywords:** intestinal immunity, vitamin, IgA, regulatory T cells, allergy, inflammation

## Abstract

The intestine is exposed continuously to complex environments created by numerous injurious and beneficial non-self antigens. The unique mucosal immune system in the intestine maintains the immunologic homeostasis between the host and the external environment. Crosstalk between immunocompetent cells and endogenous (e.g., cytokines and chemokines) as well as exogenous factors (e.g., commensal bacteria and dietary materials) achieves the vast diversity of intestinal immune functions. In addition to their vital roles as nutrients, vitamins now also are known to have immunologically crucial functions, specifically in regulating host immune responses. In this review, we focus on the immunologic functions of vitamins in regulating intestinal immune responses and their roles in moderating the fine balance between physiologic and pathologic conditions of the intestine.

## Introduction

The primary physiologic function of intestine is to serve as the chief site of nutrient absorption into the body. However, intestinal tissues also comprise a unique immune system that can discriminate between pathogens and harmless or beneficial antigens such as commensal microorganisms and dietary constituents (1). To prevent unnecessary inflammatory responses and hypersensitivity to harmless or beneficial materials, the intestinal immune system usually becomes unresponsive to these factors through the induction of oral tolerance (2). At same time, the intestinal immune system acts as the first line of defense against pathogens. For the coordinated operation of this complex network, the intestinal immune system is customized with cooperative immunocompetent cells, including the specialized antigen-sampling M cells; antigen-presenting cells [e.g., dendritic cells (DCs) and macrophages]; IgA-producing plasma cells (PCs); polarized CD4^+^ T cells such as regulatory T (T_reg_), Th1, Th2, and Th17 cells; mast cells; and innate lymphoid cells (1, 3, 4). Accumulating evidence has demonstrated that the disruption of oral tolerance underlies pathogenic conditions such as intestinal inflammation and food allergy (5).

Coordination of the numerous diverse intestinal immunological functions is achieved through the immunological crosstalk among immunocompetent cells via endogenous molecules (e.g., cytokines and chemokines). In addition to these endogenous factors, components of the gut environment, such as commensal bacteria and dietary materials, influence intestinal immunological functions. Recent advances in genetic identification have revealed that commensal bacteria play an important role in the development and maintenance of not only intestinal or mucosal immunity but also the host immune system [reviewed in Ref. (6)]. Although the underlying molecular and cellular mechanisms are not fully understood, nutritional components derived from the diet, either directly absorbed or metabolized or synthesized *de novo* by commensal bacteria, clearly are essential and influential exogenous factors for the development, maintenance, and regulation of the intestinal immune system (7, 8). This idea is underscored by the fact that nutrient deficiencies often are associated with impaired intestinal immunity (9). For instance, a recent study shows that angiotensin I converting enzyme 2 regulates intestinal amino acid metabolism and consequently affects the ecology of commensal bacteria, which leads to the transmittable colitis (10). Another recent study has demonstrated that commensal bacteria from kwashiorkor, a form of acute malnutrition that occurs by inadequate intake of dietary protein, perturb the metabolism of amino acids and carbohydrates (11).

Vitamins are organic compounds that the host organism cannot synthesize in sufficient quantities and that therefore need to be supplied exogenously by the diet or commensal bacteria. Some vitamins (e.g., vitamin B family and vitamin C) are water-soluble, whereas others (e.g., vitamins A, D, E, and K) are hydrophobic. Both hydrophilic and hydrophobic vitamins and their metabolites have diverse functions in many biologic events, including immunologic regulation. Indeed, vitamin deficiency results in high susceptibility to infection and immune diseases (12). Previously vitamins were thought to regulate the immune system in an indiscriminant manner, but accumulating evidence has revealed specific functions of individual vitamins and their metabolites in immune responses.

In this review, we discuss recent progress regarding our understanding of the immunologic functions of particular vitamins and their contributions toward maintaining the immunologic balance between physiologic and pathologic conditions of the intestine.

## Vitamin A Regulates Cell Trafficking and Differentiation in the Intestine

**Figure 1:**
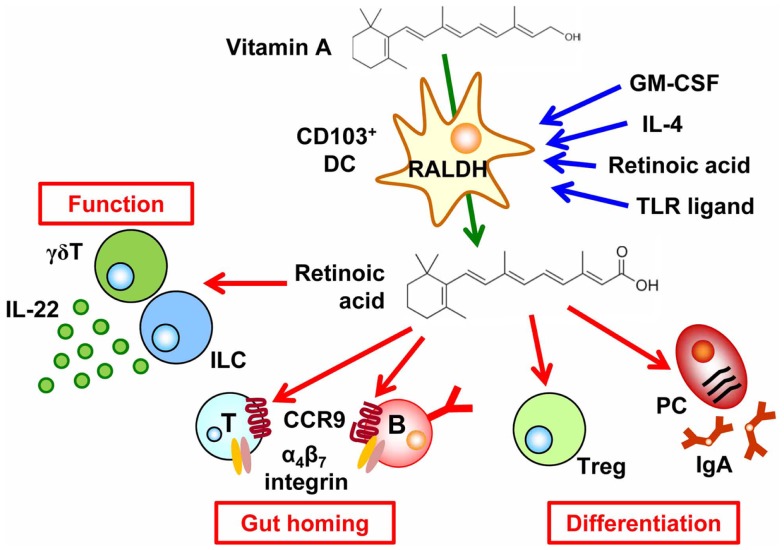
**Regulation of cell trafficking, differentiation, and function by the vitamin A metabolite retinoic acid**. CD103^+^ dendritic cells (DCs) express retinaldehyde dehydrogenases (RALDH) by GM-CSF, IL-4, TLR ligand, and retinoic acid (RA), which enable them to convert vitamin A into RA. RA then induces CCR9 and α4β7 integrin in T and B cells, causing them to migrate into the intestine. In addition, retinoic acid affects cell differentiation, such as the preferential differentiation of T cells into regulatory T (T_reg_) cells and B cells into IgA-producing plasma cells (PCs). RA also enhances IL-22 production from γδT cells and innate lymphoid cells.

Vitamin A, especially its metabolite retinoic acid (RA), has emerged as a critical mediator of mucosal immune responses [reviewed in Ref. (13)]. Vitamin A is a fat-soluble essential micronutrient obtained from diets as all-trans-retinol, retinyl esters, or β-carotene and is metabolized into retinol in tissues (14). Retinol then is converted mainly to the all-trans isoform of RA through oxidation by alcohol dehydrogenases (ADH) and retinaldehyde dehydrogenases (RALDH) (Figure 1).

The importance of vitamin A in the regulation of intestinal immunity has long been indicated. Indeed, vitamin A deficiency leads to increased susceptibility to various pathogens and vitamin A supplementation reduces the morbidity and mortality due to infectious diseases (e.g., diarrheal infections and measles) (15). During the past few years, our molecular and cellular understanding of the roles of vitamin A in the regulation of intestinal immunity has increased greatly. A key discovery was that RA regulates cell trafficking by inducing the expression of the gut-homing molecules α4β7 integrin and chemokine receptor CCR9 on lymphocytes and thus determining the gut tropism of these cells (16, 17). Epithelial cells and DCs, especially CD103^+^ DCs, in the intestine uniquely express RALDH and thus are capable of synthesizing RA; therefore the lymphocytes activated by intestinal DCs and epithelial cells express α4β7 integrin and CCR9, which allow them to return to the intestinal compartment (Figure 1). In agreement with this understanding, vitamin-A-deficient mice lack T cells and IgA-PCs in the intestine (16, 17). Several lines of evidence have demonstrated that GM-CSF induces the RALDH expression in DCs and RA itself, IL-4, and MyD88-mediated toll-like receptor pathway enhance the induction of RALDH expression (Figure 1) (18, 19).

Retinoic acid plays an important role in determining not only the gut tropism of lymphocytes activated in the intestine but also cell differentiation. For example, through the cooperative effects of TGF-β, RA promotes class switching of IgM^+^ B cells to those expressing IgA (Figure 1). Therefore, antagonism of RA results in reduced IgA production (17, 20). Another study demonstrated that Runx proteins mediate effects downstream of RA and TGF-β1 signaling in IgA class switching (21).

In addition to the effects of RA on DCs and B cells, RA affects T cell differentiation. Indeed, preferential differentiation of T cells into T_reg_ cells is mediated by CD103^+^ DCs that are capable of producing RA and activating latent TGF-β (22– 24). Reciprocally, RA failed to enhance differentiation of naïve T cells into Th17 cells in the absence of DCs (25). In this regard, DCs in the intestinal lamina propria of vitamin-A-deficient mice reportedly show impaired production of IL-6, a cytokine that is essential in the differentiation of Th17 cells (26) although there are controversial reports on the production of IL-6 by MLN-DCs from vitamin-A-deficient mice (27). On the other hand, RA–RA receptor α signal in T cells requires T cell effector responses regardless T cell subsets (26), which is in line with a previous report that Th17 cells require a low concentration of RA (20). In agreement with these functions of RA, vitamin-A-deficient mice have decreased numbers of both T_reg_ and Th17 cells in the intestine mainly due to the defect of T cell trafficking into the small intestine (25, 26, 28). In addition, segmented filamentous bacteria, Th17-inducing commensal bacteria, is decreased in vitamin A-deficient condition by high levels of mucin by goblet cells, which also leads to the impaired Th17 cell differentiation (29). Taken together, intrinsic and extrinsic factors for T cell differentiation are affected by the RA.

In addition to conventional αβ T cells, a recent study has demonstrated that RA enhanced IL-22 production by γδT cells and innate lymphoid cells, which are involved in the attenuation of intestinal inflammation (30). RA also affects non-lymphoid cells in the lymph node initiation. Indeed, RA produced by neurons adjacent to the lymph node anlagen induced CXC13 expression in stromal organizer cells and consequently led to the initial clustering of lymphoid tissue inducer cells (31). Therefore, RA has diverse functions in the regulation of versatile immunological events including cell trafficking, differentiation, cytokine production, and lymphoid organogenesis.

The various roles of RA in the mucosal immune system, especially regulating cell trafficking into the intestine, enable us to consider clinical applications of this metabolite. In general, parenteral immunization fails to achieve efficient antigen-specific immune responses in the intestine because it does not induce the necessary gut-homing molecules for the migration of antigen-sensitized immune cells into the intestine. A recent study demonstrated that the addition of RA at the time of subcutaneous vaccination increased the accumulation of antigen-specific T cells and IgA-producing PCs in the intestine and concurrently induced protective immunity against intestinal pathogens (e.g., *Salmonella*) (32). These findings suggest that exogenous RA treatment might be used to stimulate the production of gut-migrating T_reg_ cells for the control of intestinal inflammation and allergy. Additional investigation into the immune functions of RA is warranted to advance potential clinical applications of this vitamin A metabolite.

## Members of the Vitamin B Family Control Cell Metabolism and Acts as Ligands in the Regulation of Intestinal Immunity

Initially thought to be a single vitamin, vitamin B currently is recognized as a family comprising eight different members. All B vitamins are water-soluble, and they are involved in various pathways of cell metabolism. Among the B vitamins, vitamin B6 is essential for metabolism of nucleic acids, amino acids, and lipids and thus influences cell growth. Consequently, vitamin B6 deficiency leads to various impairments of immunity, such as lymphoid atrophy and reduced numbers of lymphocytes (33); conversely, vitamin B6 supplementation bolsters these weakened immune responses (34). A previous study suggested the involvement of the lipid mediator sphingosine 1-phosphate (S1P) in vitamin-B6-mediated immune regulation. S1P has been shown to regulate cell trafficking, especially cell egress from organized lymphoid tissues in both systemic (e.g., thymus, bone marrow, and lymph nodes) and mucosal (e.g., intestine) compartments [reviewed in Refs. (35, 36)]. The cell trafficking is determined by the S1P gradient that is achieved through the coordinated production of S1P and its degradation, which is mediated by S1P lyase and S1P phosphohydrolase (35). S1P lyase requires vitamin B6 as a co-factor for the degradation of S1P (37), and the administration of a vitamin B6 antagonist impair S1P lyase activity and thus create an inappropriate S1P gradient. These defects lead to impaired trafficking of lymphocytes from lymphoid tissues and consequently reduced numbers of lymphocytes in the periphery (38, 39).

Like vitamin B6, vitamin B9 (that is, folate or folic acid) is essential for nucleic acid and protein synthesis (40), and inadequate levels of vitamin B9 dramatically alter the immune response. Previous studies suggested that vitamin B9 deficiency inhibits the activity of CD8^+^ T cells and NK cells; in turn, this inhibition is associated with decreased resistance to infections (41).

**Figure 2:**
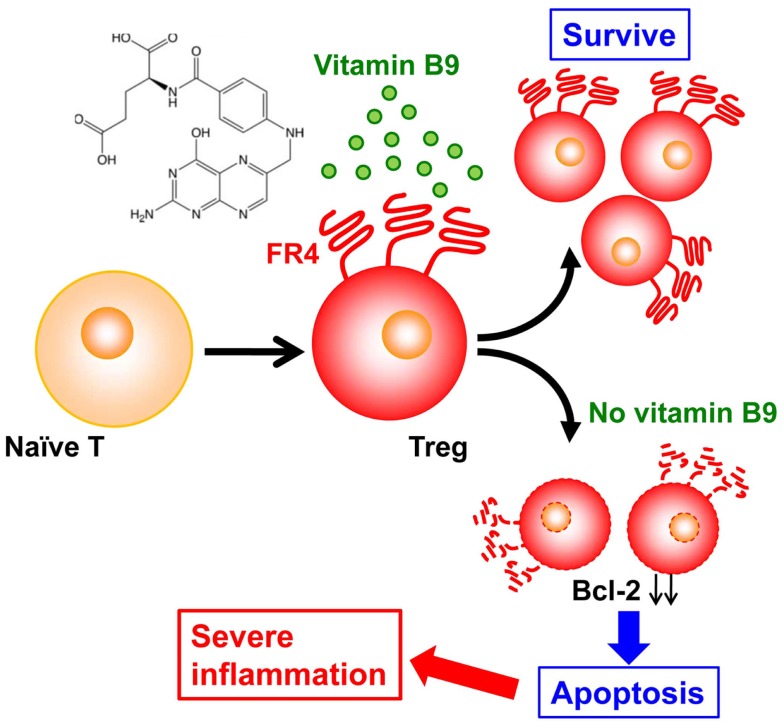
**Vitamin B9 is required for the survival of regulatory T cells and subsequent maintenance of immunologic homeostasis in the intestine**. Once naïve T cells differentiate into regulatory T (T_reg_) cells, they express folate receptor 4 (FR4), and require vitamin B9 for their survival. The absence of sufficient amounts of vitamin B9 induces the apoptosis of T_reg_ cells, with decreased expression of Bcl-2 and subsequent increased intestinal inflammation.

Folate receptor 4, a vitamin B9 receptor, is highly expressed on the surfaces of T_reg_ cells (42), implying a specific function of this vitamin in these cells. In particular, our recent study revealed that vitamin B9 is crucial in the maintenance of T_reg_ cells (43). In the absence of vitamin B9, naïve T cells can differentiate into T_reg_ cells, but differentiated T_reg_ cells fail to survive owing to the decreased expression of anti-apoptotic molecules (e.g., Bcl-2) (Figure 2). As a result, mice maintained on a vitamin-B9-deficient diet have decreased numbers of intestinal T_reg_ cells (43). As a result, the impaired survival of T_reg_ cells in these mice leads to their increased susceptibility to intestinal inflammation (Figure 2) (44).

A recent study demonstrated an additional function of the vitamin B family in the control of immune responses via mucosa-associated invariant T (MAIT) cells. MAIT cells are unconventional T cells that express a semi-invariant αβ T cell receptor that is restricted by the MHC class I-related molecule MR1; these cells are mostly found in the intestine, liver, and lung (45). Because MAIT cells can react rapidly to bacterial infections (e.g., *Escherichia coli, Klebsiella pneumoniae*, and *Mycoplasma tuberculosis*), it was supposed that the antigen presented to MR1 was bacteria-derived molecules. However, a recent study clarified that, in fact, bacterially produced metabolites of vitamin B9 and vitamin B2 bound to MR1 are presented as antigen to MAIT cells (46). Furthermore, like vitamin B2 derivatives, the vitamin B9 metabolite 6-formyl pterin binds to MR1 but, unlike vitamin B2 derivatives, fails to activate MAIT cells (46). These findings suggest that, depending on their metabolism by commensal bacteria and presentation by MR1, members of the vitamin B family can act either as positive or negative regulatory ligands for MAIT cells.

## Vitamin D is an Inhibitor of Immune Responses

**Figure 3:**
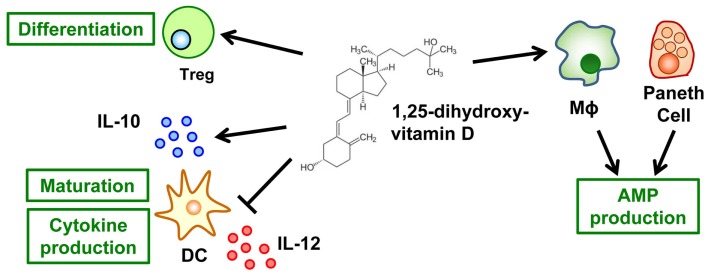
**Vitamin D mediates innate and acquired immunity**. The active form of vitamin D, it metabolite 1,25-dihydroxyvitamin D, inhibits the maturation of dendritic cells (DCs) and their production of IL-12 but simultaneously promotes their production of IL-10. In addition, T cells respond directly to 1,25-dihydroxyvitamin D for their preferential differentiation into T_reg_ cells. As a component of innate immunity, 1,25-dihydroxyvitamin D promotes the production of anti-microbial peptides (AMP) by macrophages and Paneth cells.

In its typical role of maintaining optimal concentrations of serum calcium, vitamin D is essential to a healthy mineralized skeleton (47). In addition to its effects on calcium and bone metabolism, vitamin D – especially its metabolite 1,25-dihydroxyvitamin D [1,25(OH)_2_D] – is an important regulator of the immune system, and its deficiency is linked to aberrant immune responses, including intestinal inflammation (48). Regarding a possible mechanism linking vitamin D and intestinal inflammation, 1,25(OH)_2_D may be important in the creation of an immunologic regulatory or suppressor environment. For example, 1,25(OH)_2_D inhibits the maturation of DCs and the production of their effector cytokine, IL-12, and simultaneously promotes the production of their inhibitory cytokine, IL-10, thus regulating T cell function and development (Figure 3) (49). In addition, T cells directly respond to 1,25(OH)_2_D, with preferential differentiation into T_reg_ cells (Figure 3) (50).

Furthermore, vitamin D enhances innate immunity (Figure 3). More than 25 years have passed since the anti-microbial function of 1,25(OH)_2_D against *Mycobacterium tuberculosis* in human monocytes was reported (51). Subsequent studies have revealed the molecular and cellular mechanisms underlying this anti-microbial activity. Once they are activated through Toll-like receptors, macrophages–monocytes express CYP27B1, a key enzyme in the synthesis of 1,25(OH)_2_D (52), and the vitamin D receptor (VDR) (53). These changes lead to intracrine synthesis of 1,25(OH)_2_D, which enhances the gene expression mediated by vitamin D and the VDR axis. VDR-mediated genes include the anti-microbial molecules cathelicidin (LL-37) and β-defensin 2 (54). Similar 1,25(OH)_2_D-induced production of these anti-microbial molecules occurs in epithelial cells (55) and Paneth cells (56). In addition, 1,25(OH)_2_D stabilizes tight-junction structures between epithelial cells in the intestinal tract (57). Together, these diverse functions of vitamin D contribute to the creation of the first line of defense against pathogens without the induction of aberrant inflammatory responses.

## Conclusion

Clinical evidence has long indicated that inadequate vitamin intake disrupts host immunity, thus predisposing humans to infectious and inflammatory diseases. Accumulating evidence has revealed the molecular and cellular mechanisms underlying myriad functions of vitamins in innate and acquired immune responses. These findings clarify the beneficial roles of vitamins in the maintenance of immunologic homeostasis and inform the design of vitamin analogs as pharmacologic agents for the generation and maintenance of a healthy immune condition. The complex functions of vitamins in the regulation of the immune system merit continued investigation, and these research efforts likely will enable scientists to refine our understanding of the mechanisms underlying the immunologic roles of various vitamins and to advance the development of vitamin-dependent therapeutic agents for the control of infectious and immune diseases.

## Conflict of Interest Statement

The research was conducted in the absence of any commercial or financial relationships that could be construed as a potential conflict of interest.
